# Atomically Dispersed Metal Atoms: Minimizing Interfacial Charge Transport Barrier for Efficient Carbon-Based Perovskite Solar Cells

**DOI:** 10.1007/s40820-024-01639-3

**Published:** 2025-01-31

**Authors:** Yanying Shi, Xusheng Cheng, Yudi Wang, Wenrui Li, Wenzhe Shang, Wei Liu, Wei Lu, Jiashuo Cheng, Lida Liu, Yantao Shi

**Affiliations:** 1 State Key Laboratory of Fine Chemicals, Frontier Science Center for Smart Materials, School of Chemistry, Dalian University of Technology, Dalian, 116024 People’s Republic of China; 2 School of Narcotics Control and Public Order Studies, Criminal Investigation Police, University of China, Shenyang, 110854 People’s Republic of China

**Keywords:** Perovskite solar cells, Carbon electrode, Charge transport, Energy level alignment

## Abstract

Carbon-based perovskite solar cells (C-PSCs) exhibit notable stability and durability. However, the power conversion efficiency (PCE) is significantly hindered by energy level mismatches, which result in interfacial charge transport barriers at the electrode-related interfaces. Herein, we report a back electrode that utilizes atomically dispersed metallic cobalt (Co) in carbon nanosheets (Co_1_/CN) to adjust the interfacial energy levels. The electrons in the d-orbitals of Co atoms disrupt the electronic symmetry of the carbon nanosheets (CN), inducing a redistribution of the electronic density of states that leads to a downward shift in the Fermi level and a significantly reduced interfacial energy barrier. As a result, the C-PSCs using Co_1_/CN as back electrodes achieve a notable PCE of 22.61% with exceptional long-term stability, maintaining 94.4% of their initial efficiency after 1000 h of continuous illumination without encapsulation. This work provides a promising universal method to regulate the energy level of carbon electrodes for C-PSCs and paves the way for more efficient, stable, and scalable solar technologies toward commercialization.

**Supplementary Information:**

The online version contains supplementary material available at 10.1007/s40820-024-01639-3.

## Highlights

- Atomically dispersed metal atoms effectively enhance energy level alignment and reduce energy losses at the electrode interfaces.
- The optimized carbon-based perovskite solar cells achieve a power conversion efficiency (PCE) of 22.61% and maintain 94.4% of their initial PCE after 1000 h under continuous illumination without encapsulation.

**Supplementary Information:**

The online version contains supplementary material available at 10.1007/s40820-024-01639-3.

## Graphical abstract

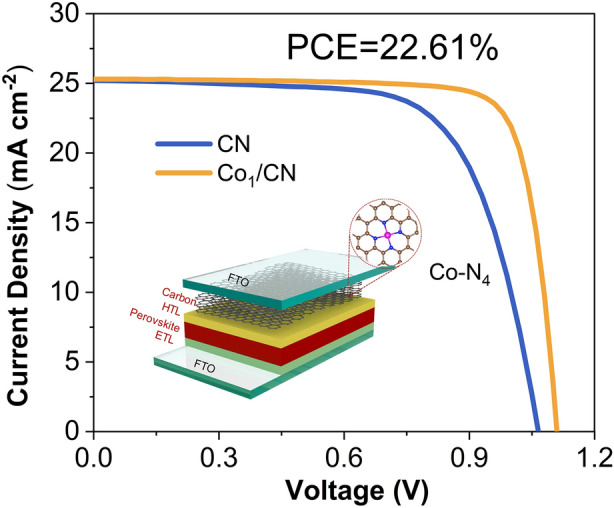

## Introduction

Despite the rapid advancements in perovskite solar cells (PSCs) efficiency, achieving high stability and durability in these devices remains a significant challenge [1–5]. In carbon-based perovskite solar cells (C-PSCs), carbon electrodes, characterized by stable C–C covalent bonds, effectively suppress undesirable reactions that undermine device stability [6–10]. For example, they mitigate the corrosion between metal electrodes and halide ions [11–14], as well as other harmful chemical reactions triggered by halide ion migration, thus preventing the degradation of the perovskite solar cells [15–18]. However, C-PSCs face substantial challenges in attaining high power conversion efficiency (PCE) comparable to their metal-based counterparts [19–22]. The main barrier arises from energy losses at the electrode interfaces, which can be attributed to three key factors: First, carbon electrodes exhibit inherently lower conductivity compared to metals, resulting in slower charge transport; second, they present a higher density of both macroscopic and microscopic defects, which notably trap and scatter charges; and third, there is an energy level mismatch between the carbon electrodes and adjacent functional layers.

Traditional doping methods that employ non-metal atoms, such as phosphorus (P), oxygen (O), and boron (B), can partially modulate the work function and conductivity of carbon materials [23–28], thus improving the performance of the device. For example, doping multi-walled carbon nanotubes with boron (B) to better align the energy levels at the perovskite/carbon interface has been shown to increase the efficiency of C-PSCs from 10.7% to 15.2% [26]. Additionally, an oxygen management strategy that increases the oxygen content in carbon black enhances energy level alignment at the perovskite/carbon interface, substantially improving hole extraction. This approach increased the open-circuit voltage (*V*_oc_) from 0.88 to 0.98 V, thereby boosting the PCE from 13.6% to 15.7% [27]. Building on these advancements, carbon-based single-atom materials (CSAMs) present even greater electrical tunability due to several factors: a wider selection of metals, more diverse and controllable coordination structures, and the rich structural diversity of the carbon substrates. Although most CSAMs have been applied in catalysis, only a few research teams have investigated their potential in solid-state optoelectronic devices [29, 30], where promising results have been observed. Nevertheless, the potential of CSAMs in the optoelectronic field remains largely unexplored and demands further development.

Hence, we present the use of CSAMs as the back electrode in C-PSCs. Specifically, atomically dispersed metallic Co is anchored to nitrogen (N) atoms on carbon nanosheets (Co_1_ /CN) in a well-defined Co-N_4_ configuration. Density functional theory (DFT) calculations reveal that the electrons in the d-orbitals of Co atoms disrupt the electronic symmetry of the carbon nanosheets (CN), inducing a redistribution of the electronic density of states that leads to a downward shift in Fermi level and a significantly reduced interfacial energy barrier, which aligns with our experimental findings. Consequently, Co_1_/CN-based devices achieved an impressive PCE of 22.61% in C-PSCs. It provides a promising universal method to improve the PCE of C-PSCs and will also enable advances in large-scale production of such materials and devices across a wide range of applications.

## Experimental Section

### Materials

All materials and reagents were obtained from commercial suppliers without further purification. The materials used included HC(NH_2_)_2_I (FAI, Greatcell Energy, Australia), CH_3_NH_3_Cl (MACl, Xi’an Yuri Solar Co., Ltd., China), lead iodide (PbI_2_, Advanced Electron Technology Co., Ltd., China), and 2,2′,7,7′-tetrakis (N,N-di-p-methoxyphenylamine)-9,9′-spirobifluorene (Spiro-OMeTAD, Borun New Material Technology Co., Ltd., China). Additionally, 4-tert-butylpyridine (TBP, TCI, Japan) was also used. Moreover, bis(trifluoromethane)sulfonimide lithium salt (Li-TFSI), anhydrous dimethylformamide (DMF), anhydrous dimethyl sulfoxide (DMSO), anhydrous chlorobenzene (CB), and anhydrous isopropyl alcohol (IPA) were all purchased from Sigma-Aldrich, USA. Fluorine-doped tin dioxide (FTO, 15 Ω sq^−1^) was custom-made by Suzhou Shangyang Solar Technology Co., Ltd., China. Glucose (from Shanghai Aladdin Biochemical Technology Co., Ltd.), melamine (from Tianjin Damao Chemical Reagent Factory), acetylacetonate cobalt (from Shanghai Aladdin Biochemical Technology Co., Ltd.), and potassium chloride (from Tianjin Damao Chemical Reagent Factory) were also used.

### Preparation of Co_1_/CN and CN

The Co_1_/CN material was synthesized using a molten salt-assisted pyrolysis method. Initially, 1.0 g of glucose and 1.0 g of melamine were used as carbon and nitrogen precursors, respectively, while 10.0 g of potassium chloride (KCl) was used as the molten salt medium. A precise amount of cobalt acetylacetonate was prepared for incorporation into the system. All solid components were combined and transferred into a ball milling jar with an appropriate amount of milling beads. The mixture was ball-milled at a frequency of 50 Hz for 5 min, repeating the process for five cycles to ensure thorough homogenization of the solid mixture. After ball milling, the homogeneous powder was separated from the milling beads and transferred to an alumina boat. The sample was placed in a tube furnace and calcined under an argon atmosphere. Calcination was conducted at 900 °C for 6 h with a controlled heating rate of 3 °C min^−1^. Once the furnace cooled to room temperature, the resulting solid underwent acid washing with 1 M hydrochloric acid (HCl) to remove residual salts and unwanted metal particles. The washed solid was filtered, dried, and collected as a black powder designated as Co_1_/CN. This method enables kilogram-scale production. For comparison, the CN sample was synthesized using the same procedure, but without the addition of cobalt acetylacetonate. This process yielded CN, the cobalt-free counterpart for subsequent analyses.

### Fabrication of Modular Co_1_/CN and CN C-PSCs

Fluorine-doped tin oxide (FTO) substrates (1.9 × 1.9 cm^2^) were initially sonicated in deionized water, ethanol, and isopropanol for at least 10 min. After cleaning, the substrate was dried at 60 °C in an oven and treated with UV-ozone for 15 min to enhance surface wettability and remove organic impurities. Next, 40 μL of SnO_2_ sol was deposited onto the conductive substrate and spin-coated at 2000 rpm for 30 s. The substrate was briefly annealed at 80 °C for 10 min. Then, 100 μL of ammonia solution (NH_3_·H_2_O) was gently added, left to stand for 10 s, and spin-coated at 2000 rpm for 20 s to remove excess ammonia. The substrate was annealed again at 80 °C for 100 min. All processes were conducted under ambient conditions (RH ≈ 30%). The perovskite layer was prepared via a two-step method. In a nitrogen-filled glove box, 1.5 M PbI_2_ was dissolved in a DMF/DMSO mixed solvent (9:1 volume ratio) and stirred at 100 °C for 30 min. Separately, 270 mg of FAI and 36 mg of MACl were dissolved in 3 mL of isopropanol (IPA) and stirred for 30 min. Both precursor solutions were filtered through 0.22 μm PTFE filters before use. The prepared SnO_2_ electron transport layer (ETL) was treated with UV-ozone for 15 min. In the nitrogen glove box, 35 μL of PbI_2_ precursor solution was spin-coated at 1500 rpm for 30 s and annealed at 70 °C for 40 s to form the PbI_2_ layer. After cooling, 100 μL of the organic salt precursor solution was spin-coated at 1700 rpm for 30 s and annealed at 90 °C for 20 s. The films were then annealed at 150 °C for 15 min under ambient conditions (RH ≈ 30%). For preparing the hole transport layer (HTL) solution, 43.38 mg of Spiro-OMeTAD was dissolved in 600 μL of chlorobenzene. Once fully dissolved, 17.5 μL of TBP and 10.5 μL of a lithium salt-acetonitrile solution (520 mg mL^−1^) were added sequentially. Then, 12 μL of Spiro-OMeTAD solution was spin-coated onto the perovskite layer to form the HTL. A carbon slurry, either based on CN or Co_1_/CN, was sprayed onto the assembled half-cell (FTO/ETL/perovskite/HTL) and the FTO substrate, followed by heating on a hot plate at 85 °C to facilitate solvent evaporation. The resulting half-cell A (FTO/ETL/perovskite/HTL/carbon) and collector B (FTO/carbon) were stacked together using retaining clips to form a modular C-PSC.

### Characterizations

X-ray diffraction (XRD) was conducted using a SmartLab 9KW diffractometer with Cu Kα X-rays (λ = 1.5406 Å) to obtain phase information. Field emission transmission electron microscopy (TEM) images were acquired using an F200 from Japan Electronics Co., Ltd. (JEOL). Inductively coupled plasma optical emission spectroscopy (ICP-OES) was performed on an Optima 2000DV from PerkinElmer. X-ray photoelectron spectroscopy (XPS) experiments were carried out using an ESCALB XI + from Thermo Fisher Scientific. High-angle annular dark-field scanning transmission electron microscopy (HAADF-STEM) and corresponding energy-dispersive spectroscopy (EDS) mapping analyses were performed on a JEOL ARM-200F(S) TEM, which is equipped with a CEOS CESCOR aberration corrector and operates at an accelerating voltage of 80 kV. The convergence semi-angle and acquisition semi-angle for ADF imaging were 28–33 and 68–280 mrad, respectively. The microstructures of the carbon electrodes were observed using a field-emission scanning electron microscope (JSM-7610F Plus, Hitachi, Japan). XPS measurements were conducted using a Kα X-ray photoelectron spectrometer (Thermo Scientific). Photoluminescence (PL) measurements were carried out using an FLS1000 spectrometer (Edinburgh Instruments, UK). The thickness of the carbon films was measured using atomic force microscopy (AFM) with a Dimension FastScan system (Bruker, USA).

### X-Ray Absorption Fine Structure Measurements

The X-ray absorption fine structure (XAFS) spectra at the Co K-edge were collected at the BL14W1 beamline of the Shanghai Synchrotron Radiation Facility (SSRF). Data acquisition was performed in fluorescence mode utilizing a Lytle detector, while corresponding reference samples were collected in transmission mode. The samples were ground and uniformly applied to a specialized adhesive tape. Hard X-rays were monochromatized using a Si(111) double-crystal monochromator, and harmonic rejection mirrors were employed for detuning to eliminate harmonics. All samples were pelletized into disks with a diameter of 8 mm, and the XAFS spectra were recorded in fluorescence mode with the Lytle detector. The acquired EXAFS data were processed following standard procedures using the ATHENA module within the IFEFFIT software package. The k^2^-weighted χ(k) data in k-space, ranging from 2.5 to 10.6 Å^−1^, were Fourier transformed into real (R) space using a Hanning window (dk = 1.0 Å^−1^) to distinguish the EXAFS contributions from different coordination shells. Effective backscattering amplitudes F(k) and phase shifts Φ(k) for all fitting paths were calculated using the ab initio code FEFF8.0.

### Characterization of Solar Cells

Electrochemical impedance spectroscopy (EIS) measurements were conducted under dark conditions utilizing an electrochemical workstation (Zennium Zahner, Germany). The current density–voltage (*J–V*) curves, which reflect the photovoltaic performance of carbon-based perovskite solar cells (C-PSCs), were obtained using a Source Measure Unit (SMU) instrument (2400, Keithley Series, USA) under AM 1.5G 1-sun illumination (100 mW cm^−2^) provided by a solar simulator (Sol3A Class AAA, Oriel, Newport, USA) in an ambient atmosphere. Before testing, the intensity of the solar light source was calibrated against an ISO-17025 standard calibrated reference silicon cell (91150 V-KG3, Newport, USA), and the active area of the device during measurements was defined using a metal mask with an area of 0.049 cm^2^. The external quantum efficiency (EQE) spectra were recorded with a quantum efficiency measurement system (QE-R, Enlitech, China). To assess the long-term operational stability of the carbon-based perovskite solar cells, a photovoltaic attenuation test system (PVLT-6001 M-16A, Suzhou D&R Instruments Co., Ltd., China) was employed, which operated under maximum power point (MPP) tracking and a continuous white LED light source (1-sun intensity) within a nitrogen glove box.

## Result and Discussion

### Synthesis and Characterization of Co_1_/CN CSAMs via Molten Salt-Mediated Pyrolysis

**Fig. 1:**
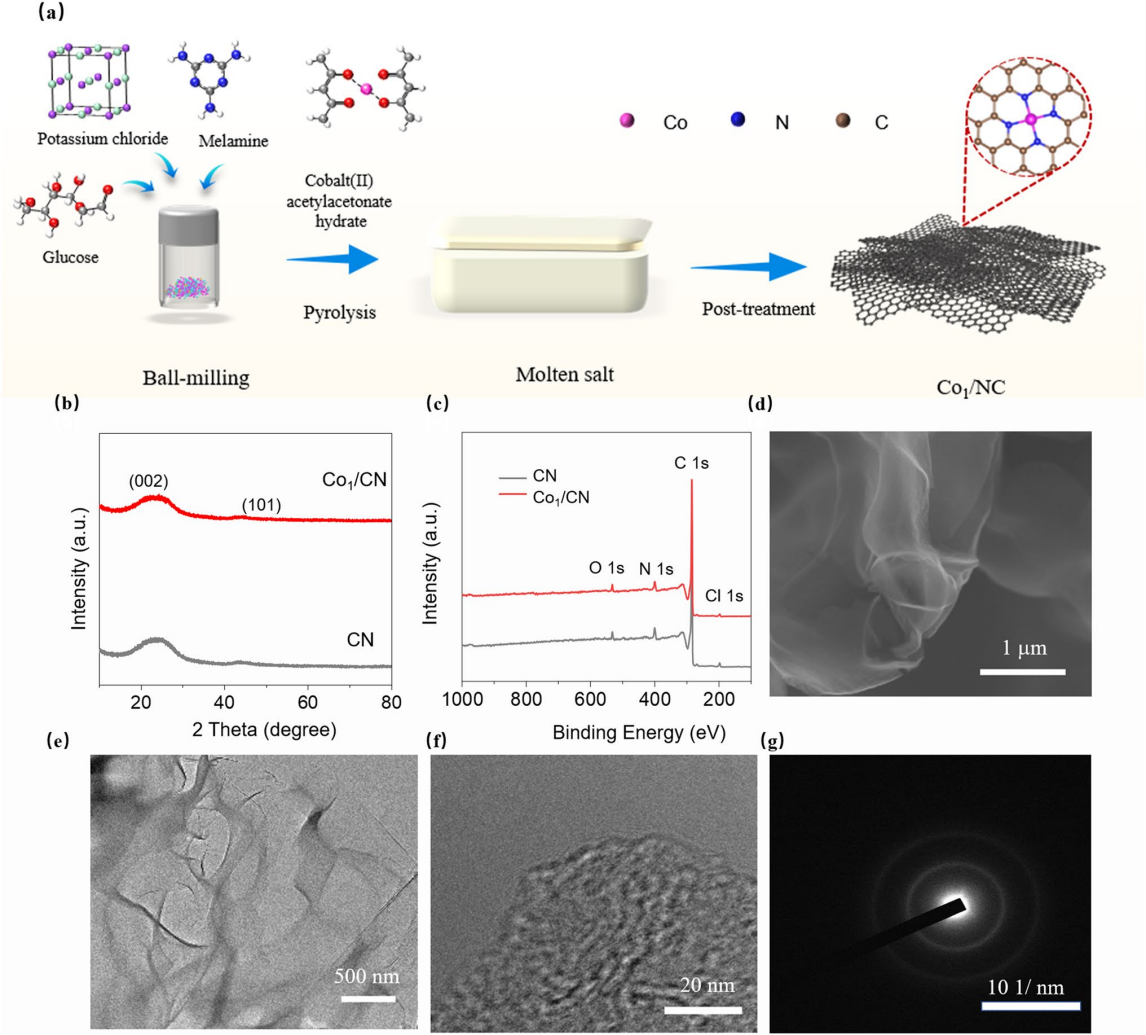
Synthesis and Characterization of Co_1_/CN CSAMs. **a** Schematic illustration of the synthesis process for Co_1_/CN via molten salt-mediated pyrolysis. **b** XRD patterns of CN and Co_1_/CN samples. **c** XPS spectra of CN and Co_1_/CN samples. **d** SEM image showing the morphology of the Co_1_/CN sample. **e, f** HRTEM image showing the morphology of the Co_1_/CN sample. **g** Selected area electron diffraction (SAED) pattern of the Co_1_/CN sample

In this work, we successfully synthesized Co_1_/CN CSAMs using a molten salt-mediated pyrolysis method. The detailed synthesis process is depicted in Fig. 1a. Specifically, melamine (C_3_H_6_N_6_) and glucose (C_6_H_12_O_6_) were used as nitrogen and carbon sources, respectively. Cobalt acetylacetonate (C_10_H_16_CoO_4_) served as the metal precursor, while potassium chloride (KCl) acted as an auxiliary medium during pyrolysis. The precursor materials were subjected to pyrolysis under an argon atmosphere in a tubular furnace. After thermal treatment, the resulting solid mixture was ground, acid-washed, filtered, and dried to yield the target sample, Co_1_/CN. A control sample (CN) was prepared using the same procedure but without the addition of the metal precursor (see Experimental Section for details).

To confirm successful synthesis and assess the structural integrity of the Co_1_/CN and CN samples, we first conducted XRD analysis, shown in Fig. 1b. Both samples exhibited characteristic diffraction peaks corresponding to graphitic carbon, with no detectable peaks from other phases [31]. The absence of additional peaks suggests that Co atoms in the Co_1_/CN material are likely present in very low concentrations and are highly anchored on the CN. Given the low Co content inferred from XRD results, we conducted an ICP-OES analysis to precisely quantify the Co concentration in Co_1_/CN (Fig. S1). The analysis revealed a Co content of 0.8 wt%, confirming the low concentration of Co and supporting the hypothesis of atomic anchored on the CN. To further investigate the elemental composition and confirm the state of Co in the samples, we conducted XPS analysis (Fig. 1c). The detailed elemental composition, determined by XPS, is presented in Table S1.

The morphology of the synthesized materials was examined by SEM (Fig. 1d). Both NC and Co_1_/CN samples exhibited a wrinkled, sheet-like morphology, with no significant pores observed on the surface; moreover, the morphology did not change substantially after Co loading. This suggests that the presence of Co does not significantly alter the overall morphology of the carbon support. The AFM image in Fig. S2 reveals that the few-layer Co_1_/CN sheet-like morphology has a randomly measured thickness of approximately 1.21 nm. TEM analysis (Fig. 1e, f) provided further evidence of the ultrathin CN, showing no large defects or pores, consistent with SEM observations. High-resolution TEM (Fig. 1f) revealed the stacking of multilayer graphene sheets, while the corresponding selected area electron diffraction (SAED) pattern (Fig. 1g) showed no crystalline diffraction spots, indicating that the material is predominantly amorphous. These results imply that Co atoms are atomically dispersed within the carbon matrix.

### Fine Structure Analysis of Co_1_/CN CSAMs

**Fig. 2:**
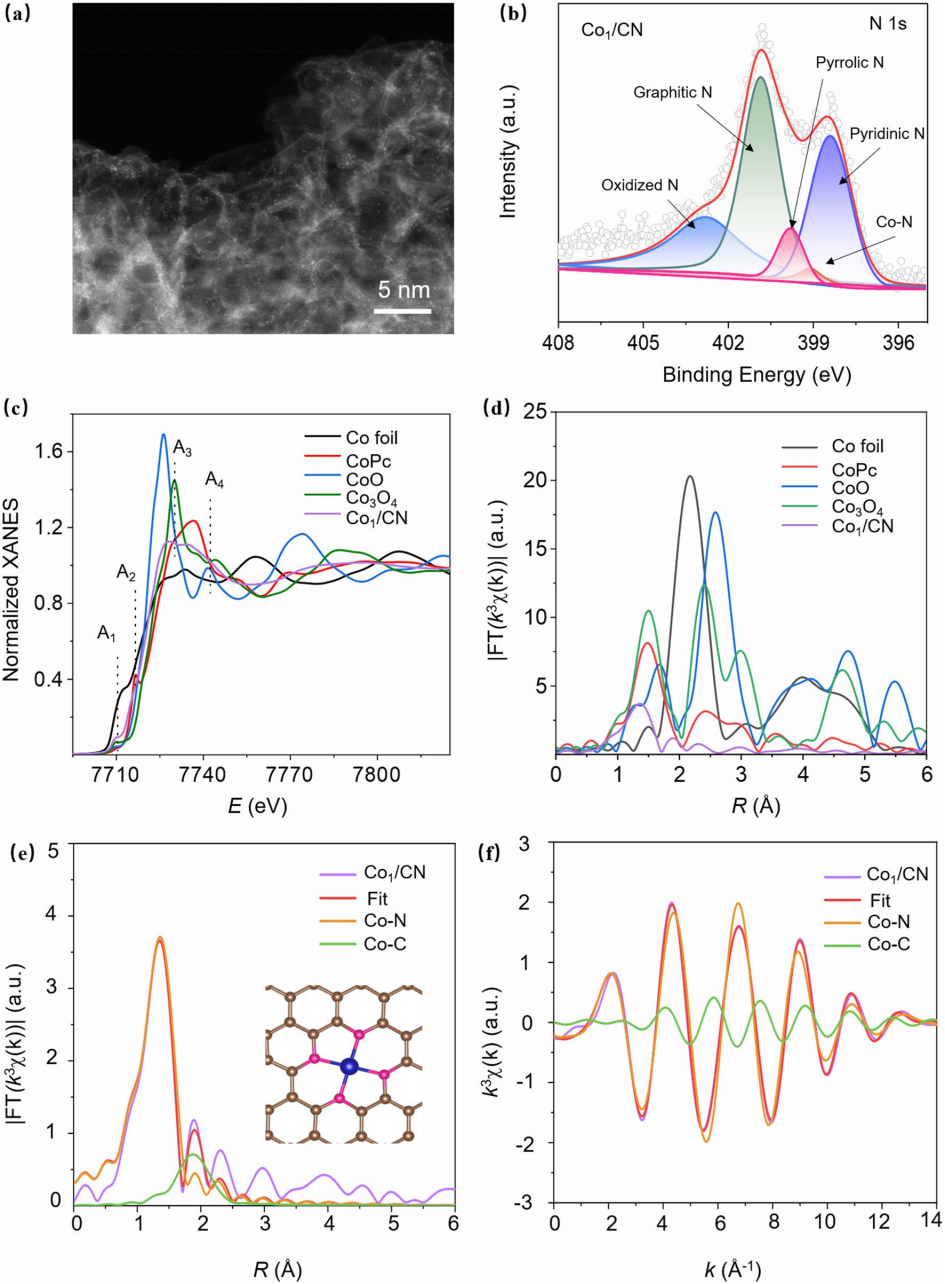
Fine Structure Analysis of Co_1_/CN CSAMs **a** HAADF-STEM image of the Co_1_/CN sample. **b** XPS N 1*s* spectrum of the Co_1_/CN sample, showing the deconvoluted peaks corresponding to various nitrogen species. **c** Normalized X-ray absorption near-edge structure (XANES) spectra at the Co K-edge for Co_1_/CN and reference samples. **d** Fourier-transformed extended X-ray absorption fine structure (FT-EXAFS) spectra at the Co K-edge for Co_1_/CN and reference samples. **e** FT-EXAFS fitting curve of the Co_1_/CN sample, with the inset showing the proposed coordination structure. **f** *k*^3^-weighted EXAFS oscillation (χ(k)) and fitting curve for the Co_1_/CN sample

The atomic dispersion of Co atoms on Co_1_/CN was confirmed by HAADF-STEM (Fig. 2a). The resulting images displayed bright spots uniformly distributed throughout the CN, which correspond to Co atoms. To further investigate the chemical states of elements, XPS analysis was conducted in the Co_1_/CN sample (Fig. 2b). The Co 2*p* spectrum of Co_1_/CN in Fig. S3 indicates the successful incorporation of Co atoms. Additionally, the deconvoluted N 1*s* spectrum revealed five distinct peaks corresponding to oxidized nitrogen (402.8 eV), graphitic nitrogen (401.2 eV), pyrrolic nitrogen (399.8 eV), pyridinic nitrogen (398.4 eV), and Co–N bonds (399.1 eV) [32–34].

X-ray absorption spectroscopy was employed to study the coordination of Co in Co_1_/CN. Figure 2c displays the X-ray absorption near-edge structure (XANES) spectra of the Co K-edge in Co foil, CoPc, CoO, and Co_1_/CN. The XANES spectra reveal distinct absorption peaks at positions A1 to A4, characteristic of Co atoms coordinated with pyridinic nitrogen. This observation suggests that the Co atoms in the Co_1_/CN sample are predominantly coordinated by nitrogen atoms in a planar configuration, similar to the coordination observed in cobalt phthalocyanine (CoPc). Furthermore, the energy position of the absorption edge in Co_1_/CN closely aligns with that of CoPc, indicating that the oxidation state of Co in the sample is primarily + 2. The Fourier-transformed extended X-ray absorption fine structure (FT-EXAFS) spectra, presented in Fig. 2d, provide additional confirmation of the local atomic structure. The pronounced peak at approximately 1.36 Å corresponds to the first coordination shell of Co–N bonds, signifying that the Co atoms are directly coordinated with nitrogen atoms in the carbon matrix. This coordination is characteristic of CSAMs, where metal atoms are stabilized by strong metal-nitrogen interactions, preventing aggregation into nanoparticles. The absence of a peak at around 2.17 Å, which would correspond to Co–Co bonds, further confirms that the Co atoms are atomically dispersed and not clustered, reinforcing the conclusion that Co_1_/CN functions as a CSAMs. Quantitative curve-fitting analysis was performed using different two-shell structural models: (a) Co–N and Co–C shell, (b) Co–N and Co–Co shell. Based on our comprehensive fittings, we found that the curve-fitting results using the structural models with and without the contribution of Co–C show a big difference. As shown in Figs. 2e, f and S4, also in Table S2, the structure model including the Co–C scatter gives the minor FT peak at about 1.90 Å (without phase shift correction). The fitted interatomic distance *R*_Co-C_ at 2.54 Å matches well with the second-shell Co–C in atomic Co_1_-N_4_C_4_ moiety [35]. In contrast, the two-shell Co–N and Co–Co structural model gives rise to rather negative (-36.6 eV) inner potential shift (Δ*E*_0_) of the Co–Co scatter, indicating that the fit is unrealistic. Therefore, the contribution from the Co–N and Co–C scatters could be evident, and we can safely conclude that a dominant majority of Co atoms are in the form of isolated Co_1_-N_4_ species in the Co_1_/CN sample.

### Electronic Properties of Co_1_/CN Electrode

**Fig. 3:**
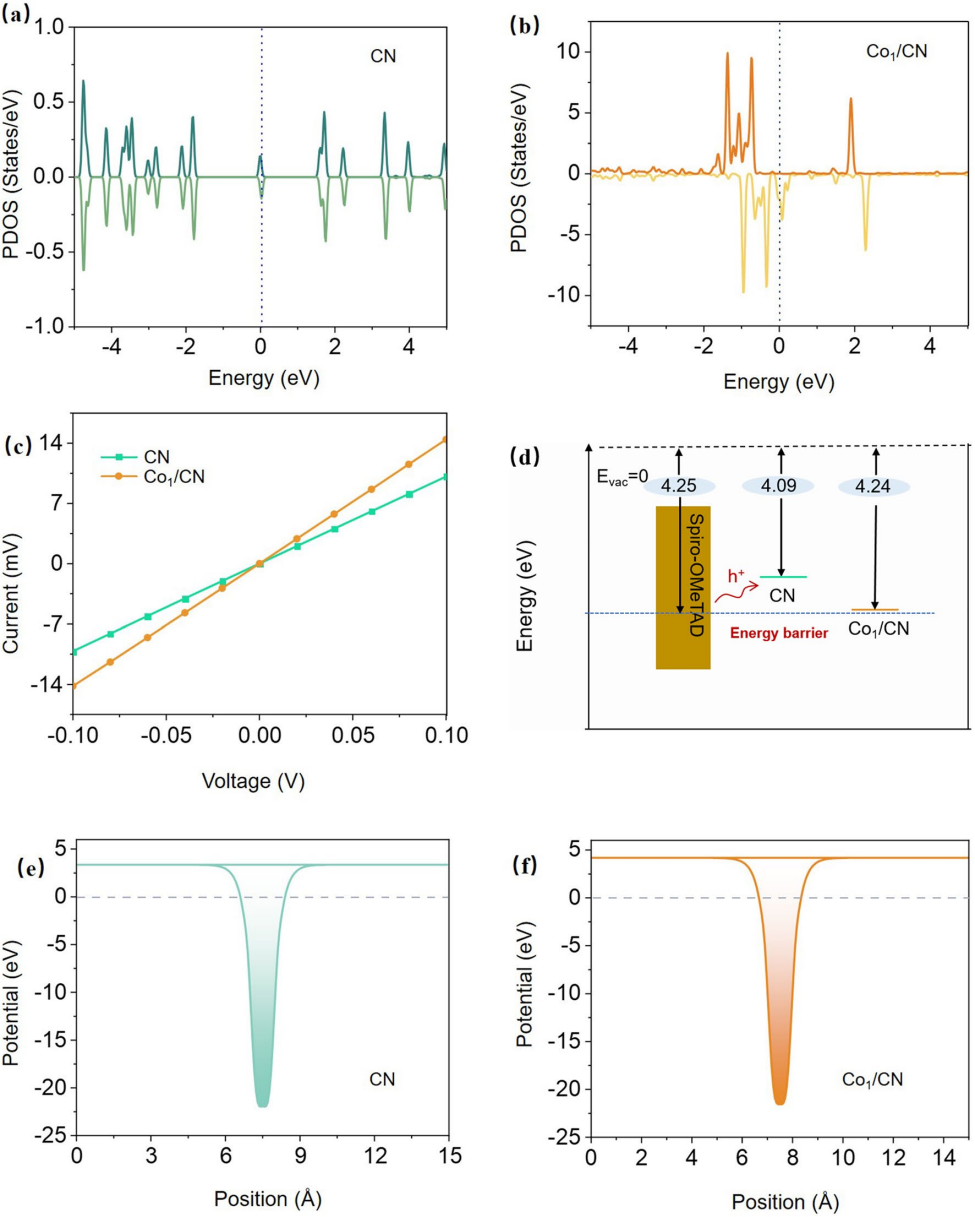
Electronic Properties of Co_1_/CN and CN samples. The partial density of states (PDOS) for **a** CN and **b** Co_1_/CN. **c** LSV characteristics of CN and Co_1_/CN. **d** Schematic representation of the energy levels. **e, f** Electrostatic potential profile for CN and Co_1_/CN along the z-direction

The electronic structures of CN and Co_1_/CN were investigated using DFT calculations. As shown by the partial density of states (PDOS) analyses in Fig. 3a, b, the significantly higher electron density of Co_1_/CN near the Fermi level compared to CN indicates higher electrical conductivity. Additionally, the electronic density of states in the Co_1_/CN system exhibits pronounced spin polarization. The electrons in the d-orbitals of Co atoms disrupt the electronic symmetry of the CN, inducing a redistribution of the electronic density of states that leads to a shift in the Fermi level. Furthermore, the band structure analysis supports these findings (Figs. S5 and S6). The band structure of undoped CN shows a distinct bandgap, reinforcing its non-metallic character. However, the introduction of Co atoms transforms the band structure, with the bandgap near the Fermi level either closing or substantially reducing, indicating a transition to metallic behavior. The electrostatic potential along the z-axis and detailed parameters were calculated for CN and Co_1_/CN. The theoretical calculations of the work function (Fig. 3e, f) show an increase from 3.37 eV for CN to 4.17 eV for Co_1_/CN. Ultraviolet photoelectron spectroscopy (UPS) analysis demonstrated an increase in the work function from 4.09 eV for CN to 4.24 eV for Co_1_/CN (Fig. S7). The energy difference between the Fermi levels of CN and Spiro-OMeTAD (Fermi level: -4.25 eV) [36] was 0.16 eV, potentially creating a significant energy barrier for charge transfer. However, Co_1_/CN reduced this difference to just 0.01 eV, effectively minimizing the energy barrier and facilitating more efficient hole transfer at the interface (Fig. 3d). These theoretical predictions are corroborated by linear sweep voltammetry (LSV) measurements, where devices with an FTO/Co_1_/CN structure exhibit significantly steeper slopes compared to those with FTO/CN (Figs. 3c and S8). This indicates that the Co_1_/CN sample shows a notably higher current density and, therefore, superior conductivity relative to undoped CN, especially at lower voltages, consistent with the DFT-calculated band structure analysis. Additionally, four-point probe resistance measurements further confirm this enhanced conductivity, as Co_1_/CN exhibits significantly lower sheet resistance compared to the CN sample. Specifically, the conductivity of Co_1_/CN (approximately 15,000 S cm^−1^) is more than twice that of CN (approximately 7,000 S cm^−1^) (Fig. S9).

### Performance Evaluation of Co_1_/CN as an Electrode Material in Perovskite Solar Cells

**Fig. 4:**
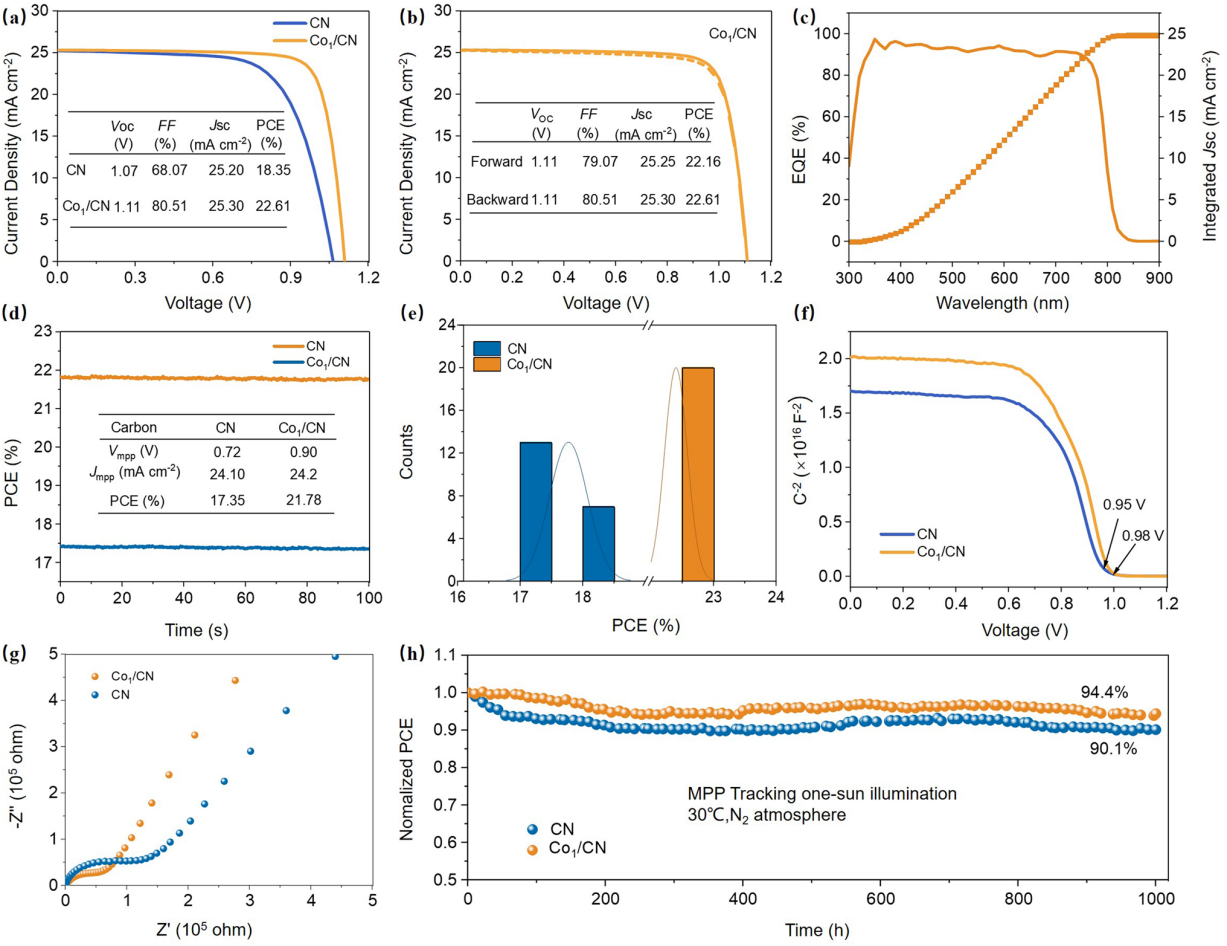
Performance evaluation of Co_1_/CN as an electrode material in perovskite solar cells. **a** Current density–voltage (*J-V*) curves of the CN and Co_1_/CN-based devices under standard one-sun illumination (AM 1.5G, 100 mW cm^−2^). **b** Forward and backward *J-V* curves of the CN and Co_1_/CN-based devices. **c** EQE spectra and the corresponding integrated *J*_sc_ for CN and Co_1_/CN-based devices, demonstrating the spectral response across different wavelengths. **d** Stability test showing the PCE over time for CN and Co_1_/CN-based devices. **e** Statistical distribution of PCE for CN and Co_1_/CN-based devices. **f** Capacitance–voltage (*C-V*) characteristics of the CN and Co_1_/CN-based devices, indicating the built-in potential (*V*_bi_) derived from the Mott–Schottky analysis. **g** Nyquist plots derived from EIS measurements for CN and Co_1_/CN-based devices. **h** Operational stability of the encapsulated C-PSC devices under continuous one-sun illumination

The photovoltaic performance of PSCs incorporating Co_1_/CN as an electrode material was systematically evaluated using n-i-p structured devices. These devices consisted of FTO/SnO_2_/Perovskite/Spiro-OMeTAD/Co_1_/CN for half-cell A and FTO/Co_1_/CN for half-cell B, stacked under controlled pressure (Fig. S10). A cross-sectional SEM image of the device interface, displayed in Fig. S11, shows the thickness and morphology of the perovskite layer on top. The CN-based devices exhibited significant enhancements, achieving a maximum PCE of 18.35%, an open-circuit voltage (*V*_oc_) of 1.07 V, a short-circuit current density (*J*_sc_) of 25.20 mA cm^−2^, and a fill factor (*FF*) of 68.07% (Fig. S12). Specifically, the optimized Co content of Co_1_/CN-based devices achieved a PCE of 22.61%, a *V*_oc_ of 1.11 V, a *J*_sc_ of 25.30 mA cm^−2^, and an *FF* of 80.51% (Figs. 4a, b and S13). We have conducted a more detailed comparative analysis of the Co_1_/CN electrodes against other common back electrode materials used in similar applications, as shown in Table S3. Furthermore, the Co_1_/CN-based devices of external quantum efficiency (EQE) measurements closely matched the *J*_sc_ values obtained from *J-V* testing (Figs. 4c and S14). Additionally, steady-state power output (SPO) measurements were conducted under continuous one-sun illumination to assess the long-term operational stability of the devices. The Co_1_/CN-based devices achieved a stabilized power output of 21.78% at their maximum power point (*V*_max_) after 100 s of illumination, significantly outperforming the control devices. This result underscores the superior stability and sustained performance of the Co_1_/CN-based devices under practical operating conditions (Fig. 4d). To ensure the reproducibility of these findings, multiple devices (20 control and 20 targets) were fabricated and tested. Statistical analysis revealed consistent improvements in *V*_oc_, *FF*, and SPO across different batches, underscoring the reliability of the performance enhancements (Figs. 4e and S15).

The improvement in *V*_oc_ is primarily attributed to the enhanced energy level alignment between the Co_1_/CN electrode and the Spiro-OMeTAD layer, as evidenced by UPS analysis. This improved alignment effectively reduces the interfacial energy barrier, thereby facilitating more efficient charge transfer and consequently increasing *V*_oc_. Photoluminescence and time-resolved PL measurements were conducted using a glass/perovskite/Spiro-OMeTAD/carbon test structure (Fig. S16). The faster charge transfer observed with Co_1_/CN electrodes can be attributed to the atomic-level dispersion of Co, enabling precise tuning of energy levels at the electrode/perovskite interface and improved energy level alignment. This reduces energy barriers and minimizes recombination at the interface, ensuring efficient charge transfer. Moreover, Mott–Schottky analysis (Fig. 4f) reveals that the built-in potential (*V*_bi_) of the control and Co_1_/CN-based devices is 0.95 and 0.98 V, respectively, indicating an enhanced driving force for carrier transfer in the treated devices. Concurrently, the enhanced conductivity of the Co_1_/CN CSAM, as demonstrated by both DFT calculations and LSV measurements, shows a significant increase in the density of states near the Fermi level and a steeper slope in the LSV curves, which leads to a reduction in series resistance and directly contributes to the improved *FF*. To analyze the impact on charge transfer dynamics, EIS was conducted on the C-PSCs, measured under dark conditions using the configuration shown in Fig. 4g at a bias of 0 V. Two distinct arcs are observed in the Nyquist plots, with their spans qualitatively reflecting the resistance associated with charge transfer and charge recombination kinetics. Compared to CN-based devices, Co_1_/CN-based devices exhibit smaller charge transfer resistance (*R*_tr_) and larger charge recombination resistance (*R*_rec_), both critical for high PCE. For quantitative assessment, Nyquist plots were fitted using a standard equivalent circuit to extract detailed impedance parameters (see Fig. S17). The Co_1_/CN-based devices demonstrate significantly lower sheet resistance *R*_s_ (from 24.50 to 22.85 Ω cm^2^), confirming enhanced conductivity of Co_1_/CN. The reduced *R*_tr_ (from 110.3 to 54.76 kΩ cm^2^) is attributed to interfacial charge transfer enabled by better energy level alignment, contributing to the observed increase in *V*_oc_. Additionally, the increased *R*_rec_ (from 5126 to 6137 kΩ cm^2^) indicates that interfacial charge recombination in the Co_1_/CN devices has been effectively suppressed, further supporting superior performance. Furthermore, dark *J-V* measurements indicate that Co_1_/CN-based devices exhibit lower dark current densities, implying better rectification and reduced leakage currents (Fig. S18). This is further corroborated by transient photovoltage (TPV) measurements under open-circuit conditions (Fig. S19), which show a prolonged carrier lifetime from 0.19 to 0.39 ms, suggesting suppressed charge carrier recombination. These improvements in leakage current suppression and charge carrier dynamics collectively contribute to achieving a higher *FF*.

These findings, supported by both theoretical and experimental analyses, underscore the effectiveness of Co_1_/CN as an advanced electrode material in significantly enhancing the overall performance of PSCs. We further investigated the impact of Co_1_/CN on the operational stability of the device, assessing stability by tracking the maximum power point (MPP) under simulated one-sun illumination (Fig. 4h). The unencapsulated Co_1_/CN-based device retained 94.4% of its initial PCE after 1000 h of continuous operation. More importantly, this study underscores the potential of CSAMs as outstanding candidates for use as electrode or interfacial layers in all-solid-state photovoltaics, likely to attract considerable interest in future research.

## Conclusions

In summary, we demonstrated a significant advancement in C-PSC through the introduction of atomically dispersed metallic Co on carbon nanosheets (Co_1_/CN) as an innovative back electrode. This approach effectively addressed the challenge of energy level mismatches that typically hinder the PCE of C-PSCs. The Co atoms interact with the electronic structure of CN, redistributing the electronic density of states and shifting the Fermi level downward. This adjustment led to a substantial reduction in the interfacial energy barrier, thereby improving charge transport at the electrode interfaces. Consequently, Co_1_/CN-based devices achieved an impressive PCE of 22.61% in C-PSCs, coupled with remarkable long-term stability. These findings highlight the potential of CSAMs to regulate the energy levels of carbon electrodes, offering a versatile and scalable solution for enhancing both efficiency and stability in C-PSCs.

## Supplementary Information

Below is the link to the electronic supplementary material.

Supplementary file1 (DOCX 5549 KB)

## References

[1] T. Liu, M.M.S. Almutairi, J. Ma, A. Stewart, Z. Xing et al., Solution-processed thin film transparent photovoltaics: Present challenges and future development. Nano-Micro Lett. **17**, 49 (2025). 10.1007/s40820-024-01547-6 10.1007/s40820-024-01547-6 PMC11499501 39441482

[2] Y. Wang, W. Li, Y. Yin, M. Wang, W. Cai et al., Defective MWCNT enabled dual interface coupling for carbon-based perovskite solar cells with efficiency exceeding 22%. Adv. Funct. Mater. **32**(31), 2204831 (2022). 10.1002/adfm.202204831

[3] H. Ma, M. Wang, Y. Wang, Q. Dong, J. Liu et al., Asymmetric organic diammonium salt buried in SnO_2_ layer enables fast carrier transfer and interfacial defects passivation for efficient perovskite solar cells. Chem. Eng. J. **442**, 136291 (2022). 10.1016/j.cej.2022.136291

[4] M. Ma, C. Zhang, Y. Ma, W. Li, Y. Wang et al., Efficient and stable perovskite solar cells and modules enabled by tailoring additive distribution according to the film growth dynamics (article). Nano-Micro Lett. **17**, 39 (2025). 10.1007/s40820-024-01538-7 10.1007/s40820-024-01538-7 PMC11480303 39404910

[5] J. Zhuang, J. Wang, F. Yan, Review on chemical stability of lead halide perovskite solar cells (review). Nano-Micro Lett. **15**, 84 (2023). 10.1007/s40820-023-01046-0 10.1007/s40820-023-01046-0 PMC10066059 37002445

[6] M. Forouzandeh, M. Heidariramsheh, H.R. Heydarnezhad, H. Nikbakht, M. Stefanelli et al., Enhanced carbon-based back contact electrodes for perovskite solar cells: Effect of carbon paste composition on performance and stability. Carbon **229**, 119450 (2024). 10.1016/j.carbon.2024.119450

[7] Y. Ren, K. Zhang, Z. Lin, X. Wei, M. Xu et al., Long-chain gemini surfactant-assisted blade coating enables large-area carbon-based perovskite solar modules with record performance. Nano-Micro Lett. **15**, 182 (2023). 10.1007/s40820-023-01155-w 10.1007/s40820-023-01155-w PMC10349030 37450089

[8] C. Dong, B. Xu, D. Liu, E.G. Moloney, F. Tan et al., Carbon-based all-inorganic perovskite solar cells: Progress, challenges and strategies toward 20% efficiency. Mater. Today **50**, 239–258 (2021). 10.1016/j.mattod.2021.05.016

[9] D. Bogachuk, S. Zouhair, K. Wojciechowski, B. Yang, V. Babu, A. Hagfeldt, A. Hinsch et al., Low-temperature carbon-based electrodes in perovskite solar cells. Energy Environ. Sci. **13**(11), 3880–3916 (2020). 10.1039/D0EE02175J

[10] J. Cheng, H. Ma, Y. Shi, L. Liu, W. Shang et al., Single-atom Ti decorated carbon black and carbon nanotubes: modular dual-carbon electrode for optimizing the charge transport kinetics of perovskite solar cells. Adv. Funct. Mater. **34**, 2409533 (2024). 10.1002/adfm.202409533

[11] S. Zhang, Y. Wang, S. Li, Z. Wang, H. Chen et al., Concerning the stability of seawater electrolysis: a corrosion mechanism study of halide on Ni-based anode. Nat. Commun. **14**(1), 4822 (2023). 10.1038/s41467-023-40563-9 37563114 10.1038/s41467-023-40563-9 PMC10415325

[12] J. Büttner, T. Berestok, S. Burger, M. Schmitt, M. Daub et al., Are halide-perovskites suitable materials for battery and solar-battery applications–fundamental reconsiderations on solubility, lithium intercalation, and photo-corrosion. Adv. Funct. Mater. **32**(49), 2206958 (2022). 10.1002/adfm.202206958

[13] D. Kong, C. Dong, X. Wei, C. Man, X. Lei et al., Size matching effect between anion vacancies and halide ions in passive film breakdown on copper. Electrochim. Acta **292**, 817–827 (2018). 10.1016/j.electacta.2018.10.004

[14] D. Li, X. Dong, P. Cheng, L. Song, Z. Wu et al., Metal halide perovskite/electrode contacts in charge-transporting-layer-free devices. Adv. Sci. **9**(36), 2203683 (2022). 10.1002/advs.202203683 10.1002/advs.202203683 PMC9798992 36319474

[15] Y. Jiang, S.-C. Yang, Q. Jeangros, S. Pisoni, T. Moser et al., Mitigation of vacuum and illumination-induced degradation in perovskite solar cells by structure engineering. Joule **4**(5), 1087–1103 (2020). 10.1016/j.joule.2020.03.017

[16] S.P. Dunfield, L. Bliss, F. Zhang, J.M. Luther, K. Zhu et al., From defects to degradation: a mechanistic understanding of degradation in perovskite solar cell devices and modules. Adv. Energy Mater. **10**(26), 1904054 (2020). 10.1002/aenm.201904054

[17] K. Domanski, E.A. Alharbi, A. Hagfeldt, M. Grätzel, W. Tress, Systematic investigation of the impact of operation conditions on the degradation behaviour of perovskite solar cells. Nat. Energy **3**(1), 61–67 (2018). 10.1038/s41560-017-0060-5

[18] N. Ahn, K. Kwak, M.S. Jang, H. Yoon, B.Y. Lee et al., Trapped charge-driven degradation of perovskite solar cells. Nat. Commun. **7**(1), 13422 (2016). 10.1038/ncomms13422 27830709 10.1038/ncomms13422 PMC5110646

[19] Q. Wang, D. Zheng, K. Wang, Q. Yang, X. Zhu et al., Versatile charge collection materials in perovskite photovoltaics. Nano Energy **128**, 109892 (2024). 10.1016/j.nanoen.2024.109892

[20] L. Perrin, E. Planes, T. Shioki, R. Tsuji, J.-C. Honore et al., How ammonium valeric acid iodide additive can lead to more efficient and stable carbon-based perovskite solar cells: Role of microstructure and interfaces? Sol. RRL **8**(17), 2400393 (2024). 10.1002/solr.202400393

[21] H. Kim, K.R. Pyun, M.-T. Lee, H.B. Lee, S.H. Ko et al., Recent advances in sustainable wearable energy devices with nanoscale materials and macroscale structures. Adv. Funct. Mater. **32**(16), 2110535 (2022). 10.1002/adfm.202110535

[22] L. Ma, Z. Bi, W. Zhang, Z. Zhang, Y. Xiao et al., Synthesis of a three-dimensional interconnected oxygen-, boron-, nitrogen-, and phosphorus tetratomic-doped porous carbon network as electrode material for the construction of a superior flexible supercapacitor. ACS Appl. Mater. Interfaces **12**(41), 46170–46180 (2020). 10.1021/acsami.0c13454 32935965 10.1021/acsami.0c13454

[23] L.K. Putri, B.-J. Ng, W.-J. Ong, H.W. Lee, W.S. Chang et al., Engineering nanoscale p–n junction via the synergetic dual-doping of p-type boron-doped graphene hybridized with n-type oxygen-doped carbon nitride for enhanced photocatalytic hydrogen evolution. J. Mater. Chem. A **6**(7), 3181–3194 (2018). 10.1039/C7TA09723A

[24] H. Hu, Z. Shi, K. Khan, R. Cao, W. Liang et al., Recent advances in doping engineering of black phosphorus. J. Mater. Chem. A **8**(11), 5421–5441 (2020). 10.1039/D0TA00416B

[25] J.P. Paraknowitsch, A. Thomas, Doping carbons beyond nitrogen: an overview of advanced heteroatom doped carbons with boron, sulphur and phosphorus for energy applications. Energy Environ. Sci. **6**(10), 2839–2855 (2013). 10.1039/C3EE4144B

[26] X. Zheng, H. Chen, Q. Li, Y. Yang, Z. Wei et al., Boron doping of multiwalled carbon nanotubes significantly enhances hole extraction in carbon-based perovskite solar cells. Nano Lett. **17**(4), 2496–2505 (2017). 10.1021/acs.nanolett.7b00200 28287749 10.1021/acs.nanolett.7b00200

[27] C. Tian, A. Mei, S. Zhang, H. Tian, S. Liu et al., Oxygen management in carbon electrode for high-performance printable perovskite solar cells. Nano Energy **53**, 160–167 (2018). 10.1016/j.nanoen.2018.08.050

[28] T. Yin, L. Long, X. Tang, M. Qiu, W. Liang et al., Advancing applications of black phosphorus and BP-analog materials in photo/electrocatalysis through structure engineering and surface modulation. Adv. Sci. **7**(19), 2001431 (2020). 10.1002/advs.202001431 10.1002/advs.202001431 PMC7539224 33042754

[29] C. Zhang, S. Liang, W. Liu, F.T. Eickemeyer, X. Cai et al., Ti_1_–graphene single-atom material for improved energy level alignment in perovskite solar cells. Nat. Energy **6**(12), 1154–1163 (2021). 10.1038/s41560-021-00944-0

[30] M. Guo, C. Wei, C. Liu, K. Zhang, H. Su et al., Composite electrode based on single-atom Ni doped graphene for planar carbon-based perovskite solar cells. Mater. Des. **209**, 109972 (2021). 10.1016/j.matdes.2021.109972

[31] Z. Wei, Y. Liu, J. Ding, Q. He, Q. Zhang et al., Promoting electrocatalytic CO_2_ reduction to CO via sulfur-doped Co-N-C single-atom catalyst. Chin. J. Chem. **41**(24), 3553–3559 (2023). 10.1002/cjoc.202300372

[32] Y. Tang, J. Chen, Z. Mao, C. Roth, D. Wang, Highly N-doped carbon with low graphitic-N content as anode material for enhanced initial Coulombic efficiency of lithium-ion batteries. Carbon Energy **5**(2), e257 (2023). 10.1002/cey2.257

[33] M. Liu, X. Zhu, Y. Song, G. Huang, J. Wei et al., Bifunctional edge-rich nitrogen doped porous carbon for activating oxygen and sulfur. Adv. Funct. Mater. **33**(11), 2213395 (2023). 10.1002/adfm.202213395

[34] P. Yu, L. Wang, F. Sun, Y. Xie, X. Liu et al., Co Nanoislands rooted on Co–N–C nanosheets as efficient oxygen electrocatalyst for Zn–air batteries. Adv. Mater. **31**(30), 1901666 (2019). 10.1002/adma.201901666 10.1002/adma.201901666 31169937

[35] H. Fei, J. Dong, Y. Feng, C.S. Allen, C. Wan et al., General synthesis and definitive structural identification of MN_4_C_4_ single-atom catalysts with tunable electrocatalytic activities. Nat. Catal. **1**(1), 63–72 (2018). 10.1038/s41929-017-0008-y

[36] Q. Zheng, F. Cao, Y. Wang, A. Tong, S. Wang et al., Synergistic effect of ionic liquid-doped spiro-OMeTAD: simultaneous management of energy level alignment and interfacial traps in perovskite solar cells. Inorg. Chem. Front. **11**(19), 6627–6637 (2024). 10.1039/D4QI01459F

