# Supplementary material for: Atomically Dispersed Metal Atoms: Minimizing Interfacial Charge Transport Barrier for Efficient Carbon-Based Perovskite Solar Cells
Source: Nanomicro Lett. 2025 Jan 31;17:125. doi: 10.1007/s40820-024-01639-3 (PMC11785869; doi:10.1007/s40820-024-01639-3)
Supplement: Supplementary file 1 — Supplementary file1 (DOCX 5549 KB) [file 40820_2024_1639_MOESM1_ESM.docx]

Supporting Information for

**Atomically Dispersed Metal Atoms: Minimizing Interfacial Charge Transport Barrier for Efficient Carbon-Based Perovskite Solar Cells**

Yanying Shi^1^, Xusheng Cheng^2^, Yudi Wang^1^*, Wenrui Li^1^, Wenzhe Shang^1^, Wei Liu^1^, Wei Lu^1^, Jiashuo Cheng^1^, Lida Liu^1^, and Yantao Shi^1^*

^1^ State Key Laboratory of Fine Chemicals, School of Chemistry, Frontier Science Center for Smart Materials, Dalian University of Technology, Dalian 116024, P. R. China

^2^ School of Narcotics Control and Public Order Studies, Criminal Investigation Police University of China, Shenyang 110854, Liaoning, P. R. China

*Corresponding authors. E-mail: [wangyd@dlut.edu.cn](mailto:wangyd@dlut.edu.cn) (Yudi Wang); [shiyantao@dlut.edu.cn](mailto:shiyantao@dlut.edu.cn) (Yantao Shi)

**Supplementary Figures**

**Fig. S1** Inductively coupled plasma optical emission spectroscopy (ICP-OES) analysis of Co content (wt%) in CN and Co_1_/CN samples

**Fig. S2** AFM image and corresponding height profiles along the blue line in d of the few-layer Co_1_/CN

**Fig. S3** XPS spectra of the Co_1_/CN samples. (**a**) Co 2*p*, (**b**) O 1*s*

**Fig. S4** FT-EXAFS fitting curve of the Co_1_/CN sample, and *k*³-weighted EXAFS oscillation (χ(k)) and fitting curve for the Co_1_/CN sample

**Fig. S5 (a)** Schematic representation of the NC structure based on DFT simulations, showing nitrogen (blue) dopant atoms embedded within the carbon (brown) lattice. **(b)** Schematic representation of the Co_1_​/CN structure based on DFT simulations, where a single cobalt atom (pink) is bonded to nitrogen atoms (blue) within the carbon lattice (brown)

**Fig. S6 (a)** Band structure and partial density of states (PDOS) of CN. The left side shows the band structure, illustrating the variation of electron energy with the wavevector, revealing the band gap. The right side presents the partial density of states for C and N atoms, where the shaded grey area represents the total density of states (DOS), and the red and blue lines correspond to contributions from carbon and nitrogen, respectively. The Fermi level is set to zero. **(b)** Band structure and partial density of states (PDOS) of Co_1_​/CN. The left side displays the band structure, with green lines indicating the contribution of cobalt atoms. The right side shows the partial density of states for C, N, and Co atoms, where the shaded grey area represents the total density of states (DOS), and the red, blue, and green lines correspond to contributions from carbon, nitrogen, and cobalt, respectively. The Fermi level is set to zero.

**Fig. S7** UPS spectra of the Co_1_/CN and CN samples

**Fig. S8** Schematic representation of the setup used for LSV testing

**Fig. S9** Electrical conductivity comparison between CN and Co_1_/CN electrodes, measured using the four-point probe method

**Fig. S10** Structural schematic diagram of Co_1_/CN and CN devices

**Fig. S11** The cross-sectional SEM images of C-PSCs using CN and Co_1_/CN electrodes

**Fig. S12** *J-V* curve of the CN device with forward and reverse scanning

**Fig. S13** The statistical distributions of PCE, *V*oc, *J*sc, and *FF* for C-PSCs using different Co content (wt%)

**Fig. S14** Incident photon-to-current efficiency spectra of CN

**Fig. S15** The statistics of photoelectric performance parameters (PCE, *J*sc, *V*oc, fill factor (*FF*)) distribution for 20 devices

**Fig. S16** PL and TRPLcurves of perovskite films with CN or Co_1_/CN

**Fig. S17** Nyquist curves of C-PSCs as well as their fitting curves and the equivalent circuit

**Fig. S18** Dark J-V curves of modular C-PSCs

**Fig. S19** TPV curves of the C-PSC devices with Co_1_/CN and CN as carbon electrode

**Table S1** Detailed elemental composition of the Co_1_/CN and CN samples as determined by X-ray photoelectron spectroscopy (XPS), highlighting the atomic-level dispersion of Co atoms, which are present in concentrations too low to be detected by XPS

| Sample | Atomic percentage (at%) | | | | |
| --- | --- | --- | --- | --- | --- |
|  | C | N | O | Cl | Co |
| CN | 90.6 | 6.08 | 2.3 | 1.02 | - |
| Co_1_/CN | 91.16 | 5.53 | 2.37 | 0.72 | 0.22 |

**Table S2** Fitting parameters of Co K-edge EXAFS curve

| **Sample** | **Path** | ***N*** | ***R* (Å)** | **σ^2^ (10^-3^Å^2^)** | **Δ*E*_0_ (eV)** | ***R*-factor** |
| --- | --- | --- | --- | --- | --- | --- |
| sample | Co-N | 3.6±0.4 | 1.86±0.01 | 7.2±1.1 | -3.3±1.2 | 0.001 |
|  | Co-C | 2.0±0.4 | 2.55±0.02 |  |  |  |
| sample | Co-N | 4.1±0.4 | 1.87±0.01 | 8.4±1.2 | -0.9±1.2 | 0.001 |
|  | Co-Co | 0.5±0.2 | 2.42±0.02 |  | -36.6±1.9 |  |

**Table S3** Comparison of photovoltaic parameters against other common back electrode materials

| **Common back electrode materials** | ***V_OC_***  **(V)** | ***J_SC_***  **（mA cm^-2^）** | ***FF***  **(%)** | **PCE**  **(%)** | **Work function (eV)** | **Refs.** |
| --- | --- | --- | --- | --- | --- | --- |
| Carbon paste | 1.05 | 23.50 | 79.10 | 19.52 | / | [S1] |
| Carbon paste | 1.14 | 24.30 | 68.90 | 19.00 | 5.0 | [S2] |
| Carbon paste | 1.02 | 25.00 | 79.00 | 20.07 | 5.0 | [S3] |
| Carbon paste | 1.13 | 20.47 | 79.00 | 18.27 | 5.0 | [S4] |
| Carbon paste | 1.11 | 23.70 | 76.00 | 19.20 | 5.0 | [S5] |
| Carbon paste | 1.19 | 18.45 | 81.56 | 17.88 | 5.0 | [S6] |
| Carbon paste | 1.13 | 24.00 | 78.00 | 21.20 | / | [S7] |
| Carbon paste | 1.14 | 20.40 | 81.64 | 19.08 | 5.0 | [S8] |
| Carbon paste | 1.06 | 25.60 | 82.00 | 22.20 | / | [S9] |
| Carbon paste | 1.61 | 7.82 | 82.46 | 10.40 | 5.0 | [S10] |
| Carbon paste | 1.08 | 24.16 | 78.00 | 20.41 | 5.0 | [S11] |
| Carbon paste | 1.13 | 19.84 | 82.30 | 18.48 | / | [S12] |
| Carbon paste | 1.09 | 24.10 | 77.60 | 20.40 | 5.0 | [S13] |
| Carbon paste | 1.13 | 22.95 | 74.20 | 19.30 | 5.0 | [S14] |
| Carbon paste | 1.08 | 18.70 | 76.40 | 15.35 | / | [S15] |
| Carbon paste | 1.07 | 21.34 | 67.29 | 15.40 | / | [S16] |
| Carbon paste | 1.19 | 14.20 | 76.21 | 12.89 | 5.0 | [S17] |
| Carbon paste | 1.09 | 23.46 | 74.7 | 19.10 | / | [S18] |
| Carbon black | 1.09 | 24.66 | 72.10 | 19.41 | / | [S19] |
| Carbon black | 1.00 | 24.60 | 82.00 | 20.09 | / | [S20] |
| Graphene | 1.08 | 25.90 | 78.00 | 22.07 | 4.31 | [S21] |
| Co_1_/CN | 1.11 | 25.30 | 80.51 | 22.61 | 4.24 | Our work |

**Supplementary References**

1. L. Li, H. Rao, Z. Wu, J. Hong, J. Zhang et al., Moisture induced secondary crystal growth boosting the efficiency of hole transport layer-free carbon-based perovskite solar cells beyond 19.5%. Adv. Funct. Mater. **34**, 2308428 (2024). <https://doi.org/10.1002/adfm.202308428>
2. N. Cheng, W. Li, H. Pan, D. Zheng, W.-X. Yang et al., Promote the performance of carbon electrode-based perovskite solar cells with Cu_2_GeS_3_ hole transporting layer. Chem. Eng. J. **489**, 151463 (2024). <https://doi.org/10.1016/j.cej.2024.151463>
3. C. Han, J. Du, Z. Liu, Q. Gao, X. Chen et al., In situ reconstruction post-treatment for efficient carbon-based hole-conductor-free printable mesoscopic perovskite solar cells. Adv. Funct. Mater. **34**, 2408686 (2024). <https://doi.org/10.1002/adfm.202408686>
4. H. Wang, Q. Zhang, Z. Lin, H. Liu, X. Wei et al., Spatially selective defect management of CsPbI_3_ films for high-performance carbon-based inorganic perovskite solar cells. Sci. Bull. **69**, 1050-1060 (2024). <https://doi.org/10.1016/j.scib.2024.01.0387>
5. T. Du, S. Qiu, X. Zhou, V. M. Le Corre, M. Wu et al., Efficient, stable, and fully printed carbon-electrode perovskite solar cells enabled by hole-transporting bilayers. Joule **7**, 1920-1937 (2023). <https://doi.org/10.1016/j.joule.2023.06.005>
6. X. Huo, J. Lv, K. Wang, W. Sun, W. Liu et al., Surface sulfidation constructing gradient heterojunctions for high-efficiency (approaching 18%) HTL-free carbon-based inorganic perovskite solar cells. Carbon Energy, e586 (2024). <https://doi.org/10.1002/cey.20240586>
7. L. Luo, H. Zeng, Z. Wang, M. Li, S. You et al., Stabilization of 3D/2D perovskite heterostructures via inhibition of ion diffusion by cross-linked polymers for solar cells with improved performance. Nat. Energy **8**, 294-303 (2023). <https://doi.org/10.1038/s41560-023-01205-y>
8. J. Lin, R. Huang, X. Peng, J. Zhang, G. Zhang et al., Eliminating hole extraction barrier in 1D/3D perovskite heterojunction for efficient and stable carbon-based CsPbI3 solar cells with a record efficiency. Adv. Mater. **36**, 2404561 (2024). <https://doi.org/10.1002/adma.202404561>
9. J. Liu, X. Chen, K. Chen, W. Tian, Y. Sheng et al., Electron injection and defect passivation for high-efficiency mesoporous perovskite solar cells. Science **383**, 1198-1204 (2024). <https://doi.org/10.1126/science.adk9089>
10. Z. Wang, B. He, M. Wei, W. Liu, X. Li et al., Enhanced charge extraction enabled by amide-functionalized carbon quantum dots modifier for efficient carbon-based perovskite solar cells. Chem. Eng. J. **479**, 147736 (2024). <https://doi.org/10.1016/j.cej.2023.147736>
11. J. Hong, C. Kang, R. Huang, Z. Wu, L. Li et al., Anethole regulated crystallization for high-efficiency carbon-based perovskite solar cells. Adv. Funct. Mater. **34**, 2405374 (2024). <https://doi.org/10.1002/adfm.202405374>
12. W. Wang, X. Peng, J. Zhang, J. Lin, R. Huang et al., Dimethylamine oxalate manipulating CsPbI3 perovskite film crystallization process for high-efficiency carbon electrode-based perovskite solar cells. Energy Chem. **93**, 221-228 (2024). <https://doi.org/10.1016/j.jechem.2024.01.059>
13. J. W. Tang, Y. Lin, H. Yan, J. Lin, H. Rao et al., 20.1% certified efficiency of planar hole transport layer-free carbon-based perovskite solar cells by spacer cation chain length engineering of 2D perovskite. Angew. Chem. Int. Ed. **63**, e202406167 (2024). <https://doi.org/10.1002/anie.202406167>
14. T. Ye, Y. Hou, A. Nozariasbmarz, D. Yang, J. Yoon et al., Cost-effective high-performance charge-carrier-transport-layer-free perovskite solar cells achieved by suppressing ion migration. ACS Energy Lett. **6**, 3044-3052 (2021). <https://doi.org/10.1021/acsenergylett.1c01186>
15. H. Wang, H. Liu, Z. Dong, X. Wei, Y. Song et al., Extracting ammonium halides by solvent from the hybrid perovskites with various dimensions to promote the crystallization of CsPbI_3_ perovskite. Nano Energy **94**, 106925 (2022). <https://doi.org/10.1016/j.nanoen.2022.106925>
16. F. Yang, L. Dong, D. Jang, K. C. Tam, K. Zhang et al., Fully solution-processed pure α-phase formamidinium lead iodide perovskite solar cells for scalable production in ambient condition. Adv. Energy Mater. **10**, 2001869 (2020). <https://doi.org/10.1002/aenm.202001869>
17. L. Wang, B. Fan, D. Wei, Z. Yang, B. Zheng et al., Efficient carbon-based CsPbI_2_Br perovskite solar cells using bifunctional polymer modification. Sustain. Energy Fuels **5**, 3867-3875 (2021). <https://doi.org/10.1039/D1SE00713K>
18. H. Zhang, Y. Li, S. Tan, Z. Chen, K. Song et al., High-efficiency (>20%) planar carbon-based perovskite solar cells through device configuration engineering. J. Colloid Interface Sci. **608**, 3151-3158 (2022). <https://doi.org/10.1016/j.jcis.2021.11.050>
19. J. Ma, S. Lin, M. Fang, Z. Fang, X. Yu et al., Octylammonium iodide induced in-situ healing behavior at the perovskite-carbon interface: the “slow-release effect” caused by carbon black adsorption. Small **20**, 2310196 (2024). <https://doi.org/10.1002/smll.202310196>
20. D. A. Chalkias, A. Nikolakopoulou, L. C. Kontaxis, A. N. Kalarakis, E. Stathatos, Record-breaking efficient and mechanically robust ambient-air-processed carbon-based flexible perovskite photovoltaics through effective and benign-to-plastics green-antisolvent quenching. Adv. Funct. Mater. **34**, 2406354 (2024). <https://doi.org/10.1002/adfm.202406354>
21. Y. Wang, W. Li, Y. Yin, M. Wang, W. Cai et al., Defective MWCNT enabled dual interface coupling for carbon-based perovskite solar cells with efficiency exceeding 22%. Adv. Funct. Mater. **32**, 2204831 (2022). <https://doi.org/10.1002/adfm.202204831>
